# Evaluation of Molecular Methods To Improve the Detection of Burkholderia pseudomallei in Soil and Water Samples from Laos

**DOI:** 10.1128/AEM.04204-14

**Published:** 2015-05-05

**Authors:** Michael Knappik, David A. B. Dance, Sayaphet Rattanavong, Alain Pierret, Olivier Ribolzi, Viengmon Davong, Joy Silisouk, Manivanh Vongsouvath, Paul N. Newton, Sabine Dittrich

**Affiliations:** a Lao-Oxford-Mahosot Hospital-Wellcome Trust Research Unit, Microbiology Laboratory, Mahosot Hospital, Vientiane, Lao People's Democratic Republic; b Centre for Tropical Medicine and Global Health, Nuffield Department of Medicine, University of Oxford, Oxford, England, United Kingdom; c Institute of Ecology and Environmental Science—Paris, Institut de Recherche pour le Développement (IRD), Vientiane, Lao People's Democratic Republic; d Géosciences Environnement Toulouse (GET), UMR 5563, IRD, Université de Toulouse, UPS (OMP), CNRS, Toulouse, France

## Abstract

Burkholderia pseudomallei is the cause of melioidosis, a severe and potentially fatal disease of humans and animals. It is endemic in northern Australia and Southeast Asia and is found in soil and surface water. The environmental distribution of B. pseudomallei worldwide and within countries where it is endemic, such as the Lao People's Democratic Republic (Laos), remains unclear. However, this knowledge is important to our understanding of the ecology and epidemiology of B. pseudomallei and to facilitate public health interventions. Sensitive and specific methods to detect B. pseudomallei in environmental samples are therefore needed. The aim of this study was to compare molecular and culture-based methods for the detection of B. pseudomallei in soil and surface water in order to identify the optimal approach for future environmental studies in Laos. Molecular detection by quantitative real-time PCR (qPCR) was attempted after DNA extraction directly from soil or water samples or after an overnight enrichment step. The positivity rates obtained by qPCR were compared to those obtained by different culture techniques. The rate of detection from soil samples by qPCR following culture enrichment was significantly higher (84/100) than that by individual culture methods and all culture methods combined (44/100; *P* < 0.001). Similarly, qPCR following enrichment was the most sensitive method for filtered river water compared with the sensitivity of the individual methods and all individual methods combined. In conclusion, molecular detection following an enrichment step has proven to be a sensitive and reliable approach for B. pseudomallei detection in Lao environmental samples and is recommended as the preferred method for future surveys.

## INTRODUCTION

The Gram-negative bacterial saprophyte Burkholderia pseudomallei is the causative agent of melioidosis and is found in soil and surface water predominantly in regions of Southeast Asia and northern Australia, where the organism is endemic (1–3). It is a common cause of fatal community-acquired bacteremia, pneumonia, and visceral and soft tissue abscesses and poses a significant public health burden (4, 5). Most patients are thought to contract the infection from skin inoculation; other possible routes of transmission are inhalation and ingestion (6). Due to the high mortality from infection with the organism and potential transmission of the organism by aerosols, B. pseudomallei is classified as a tier 1 select agent (7). Melioidosis was first reported in the Lao People's Democratic Republic (Laos) in 2001 with the description of two patients with the disease (8). In a randomized soil survey conducted in 2009, the highest isolation frequency was in Saravane Province (southern Laos) (9). Little is known about the true geographical distribution of melioidosis across the country, highlighting the need for a detailed risk map to support empirical patient management. The current “gold standard” for detection of environmental B. pseudomallei is culture from soil or water samples (10, 11). Culture methods for soil samples have successfully been employed in many studies, with proposed consensus guidelines being based on a simplified qualitative technique recently published (11). Similar guidelines do not yet exist for the detection of B. pseudomallei in water (3, 11, 12). Investigations of B. pseudomallei in Laos have highlighted the challenges of detecting the pathogen in different environmental samples (9, 12), hampering the development of a detailed risk map (9). Despite their extensive use, culture methods have their limitations, including the potential overgrowth of B. pseudomallei by other environmental bacterial species, especially when it is present in small numbers. In addition, bacteria in a viable but noncultivable state are not detectable (13), further decreasing the sensitivity of this approach.

To overcome these limitations, molecular methods have successfully been developed and applied to detect B. pseudomallei in soil (14, 15). A study in Thailand demonstrated that molecular detection estimated the bacterial load per gram of soil to be 10 times higher than that determined by culture methods (15), and investigations from northern Australia showed that quantitative real-time PCR (qPCR) following an enrichment step yielded a higher positivity rate than culture alone (14). Furthermore, the high specificity of molecular assays allows the specific detection of B. pseudomallei even if closely related bacteria, such as Burkholderia thailandensis, Burkholderia vietnamiensis, Burkholderia cepacia, and other Burkholderia and Ralstonia species, are present in the same environment (16–18). Although molecular methods have been used for the detection of B. pseudomallei from soil, no molecular methods for the detection of the pathogen directly from water samples have been described. As soil and water sampling is crucial for mapping disease risks in the country, updated techniques suitable for large-scale screening are urgently needed.

The aim of this study was to use described and novel molecular methods for the detection of B. pseudomallei in soil and water and evaluate those approaches in comparison to different culture techniques.

## MATERIALS AND METHODS

### Sample collection. (i) Soil.

**FIG 1:**
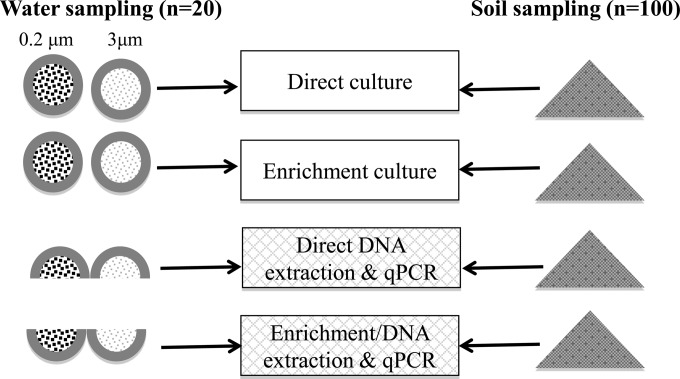
Schematic representation of the methodologies and environmental samples used in the study. Soil samples were taken from two different depths (30 cm and 60 cm; ∼0.5 g for DS DNA extraction, ∼10 g for simplified culture, ∼20 g for ES DNA extraction, ∼100 g for conventional culture) at 50 positions in the rice field, and all samples were processed by direct or enrichment culture, direct DNA extraction, or DNA extraction postenrichment. Water samples were collected at 20 different sites, separate samples were filtered through filters of two different pore sizes (total *n* = 40), and all filters were processed by direct and enrichment culture, while filters were split for molecular methods (direct DNA extraction and DNA extraction postenrichment). In addition, 10 Moore's swabs were collected, and the water from these was cultured directly on solid medium (not shown), as previously described (12). The latitudes/longitudes of the sampling sites were as follows: 15°7′26.22″N/105°48′28.02″E, 15°21′38.81″N/105°49′52.85″E, 15°34′48.01″N/105°48′42.50″E, 15°40′4.62″N/105°54′40.13″E, 15°42′31.58″N/106°4′7.87″E, 15°47′23.56″N/106°17′26.87″E, 15°42′41.46″N/106°25′41.95″E, 15°13′16.88″N/105°44′34.96″E, 15°20′5.86″N/105°58′58.87″E, 15°15′30.33″N/105°55′59.81″E, 15°29′44.72″N/105°45′51.05″E, 15°41′44.84″N/106°16′1.23″E, 15°38′12.39″N/106°22′3.86″E, 15°39′16.84″N/105°50′54.55″E, 15°42′0.52″N/105°58′30.19″ E, 15°42′26.27′N/106°8′38.07″E, 15°40′52.12″N/106°25′59.19″E, 15°32′24.51″N/106°15′54.90″E, 15°27′59.66″N/106°10′12.32″E, 15°24′33.56″N/106°5′27.98″E.

Soil sampling was performed during the dry season (April 2013) in a rice paddy near the village of Ban Nabone, Vientiane Province, Laos (18°22′51.4″N, 102°25′27.8″E; altitude, 195 m). Samples were collected from two depths (30 cm and 60 cm) at 50 random points within a section of the field previously determined to have the highest rates of positivity for B. pseudomallei by culture (19), with a minimum distance of 2 m being allowed between sampling sites. Oral informed consent for the removal of soil samples was obtained from the farmers concerned, and written permission was obtained from the relevant authorities. Samples were collected from the field using a hand auger that was disinfected between samplings with 70% alcohol (9). The samples were placed in sterile plastic bags, which were placed in an insulated box that was kept in the shade and maintained at ambient temperature during transport and subsequent manipulation. To ensure that representative subsamples were obtained, the two-dimensional Japanese slab cake method was used, and ∼0.5-g (direct extraction from soil), ∼10-g (simplified culture method [11]), ∼20-g (extraction postenrichment [14]), and ∼100-g (conventional culture method [20]) subsamples were obtained (Fig. 1) (21, 22).

Samples were collected within 24 h of each other, and subsampling was performed up to 72 h postsampling. Processing of all samples was started on the same day at 120 h postsampling.

### (ii) Water.

Water samples (600 ml; *n* = 20) were collected along the course of the Sedone River (Saravane Province, Laos) (*n* = 7) and its main tributaries (*n* = 13) at the onset of the rainy season (June 2013; mean turbidity, 257 nephelometric turbidity units; mean water temperature, 26.3°C; mean pH, 7.1; see Table S2 in the supplemental material). Collection bottles were triple rinsed with river water from the collection site before collecting the sample. The water was mixed thoroughly, and 30-ml subsamples were filtered within 8 h of collection, using 47-mm-diameter membranes. Six subsamples of water were filtered from each site; three were filtered using 0.2-μm-pore-size filters (30 ml, cellulose acetate membrane filter; Sartorius), and three were filtered using 3.0-μm-pore-size filters (cellulose nitrate membrane filter; Sartorius) in an attempt to estimate total and attached bacterial loads, respectively (Fig. 1) (23, 24). A manual pump and a 1-liter glass vacuum flask with a stainless steel funnel were used, and membrane supports were sterilized with 70% ethanol between filtrations. In addition, Moore's swab samples (*n* = 10) were collected as described previously (12).

### Culture techniques. (i) Soil.

Soil was cultured using both plating of soil suspension supernatants on Ashdown's agar as previously described (20) and the simplified broth enrichment culture method recommended in recent guidelines (11). The results of this comparison (unpublished data) will be reported elsewhere.

### (ii) Water.

Three methods were used concurrently to attempt to culture B. pseudomallei from water at each sampling site. First, Moore's swabs (*n* = 10) were used as described previously (12). Second, individual filters from each sampling point (*n* = 40; Fig. 1) were placed in 10 ml modified Ashdown's broth (14) and incubated aerobically without shaking for 7 days at 40°C. At days 3 and 7, 10-μl and 100-μl samples of broth were subcultured onto Ashdown's agar containing gentamicin at 8 mg/liter and incubated at 40°C aerobically for 4 days with daily inspections for colonies resembling B. pseudomallei (12). Third, filters from each sampling point (*n* = 40; Fig. 1) were placed directly on Ashdown's agar plates and cultured at 40°C for up to 96 h. B. pseudomallei was identified and confirmed as described previously (12). Briefly, all positive cultures were screened by agglutination with a latex agglutination reagent specific for the 200-kDa extracellular polysaccharide of B. pseudomallei and tested for susceptibility to amoxicillin-clavulanic acid (co-amoxiclav) and resistance to colistin (8, 14). All suspected isolates were confirmed to be B. pseudomallei by qPCR (16), and selected isolates were confirmed to be B. pseudomallei by use of an API 20NE system.

### Molecular detection. (i) Soil.

Direct soil (DS) DNA extraction, in which DNA was extracted from ∼0.5 g of soil, was attempted using a kit (PowerSoil DNA isolation kit; Mo Bio) as described previously (14). Enrichment soil (ES) DNA extraction was done as described previously (14) with minor modifications. In brief, soil was homogenized in the modified Ashdown's enrichment broth, shaken for 2 h at 240 rpm, and then incubated at 37°C for 22 h. The liquid phase (∼10 ml) was decanted and centrifuged at 700 × *g* for 2 min, and the supernatant (∼7 ml) was removed and aurintricarboxylic acid (14) was added. After further centrifugation (45 min, 4,000 × *g*), DNA was extracted from the soil pellet (∼0.5 g) as described previously (14).

### (ii) Water.

Filters were cut diametrically in half; one half was used to extract DNA directly from the filter (direct filter [DF] extraction), while the other half was subjected to an enrichment step (enrichment filter [EF] extraction) (Fig. 1). For DF extraction, the filter membrane was cut into small pieces using sterile scissors. DNA was extracted from the filter membranes with the PowerSoil DNA isolation kit (Mo Bio), and the detachment of soil particles could be visually observed. After 60 μl of C1 solution was added, DNA extraction was performed according to the manufacturer's instructions (Mo Bio). The second half, used for EF extraction, was incubated in modified Ashdown's broth for 24 h at 37°C (14). Membranes were repeatedly vortexed to release the sediment and bacteria, and after incubation, the filters were removed and free sediment and bacteria were pelleted (45 min, 4,000 × *g*). The pellet (∼0.2 g) was processed for DNA extraction as described previously (14).

### qPCR.

The PCR targets a 115-bp stretch in *orf2* of the type III secretion system gene cluster (TTS1) of B. pseudomallei and was performed, with minor modifications, as described previously (14, 16). In brief, the 25-μl master mix consisted of 1 U Platinum *Taq* DNA polymerase (Invitrogen), 500 nM each primer, 250 nM probe, 7 mM MgCl_2_, 1× PCR buffer, 200 μM deoxynucleoside triphosphates, and 4 μl of soil DNA. To reduce the effect of inhibitors, 400 ng/μl of bovine serum albumin (BSA; New England Biolabs, USA) was added (15). To control for PCR inhibitors, ∼10^5^ copies of inhibitor control plasmid (47 kDa, Orientia tsutsugamushi [25, 26]) were amplified alone and in parallel spiked with 4 μl of sample DNA. Inhibition was monitored as described previously (14). Amplification was performed on a Rotor-Gene 6000 system (Qiagen, Germany) at 94°C for 10 min, and then 45 cycles of 94°C for 15 s and 60°C for 1 min were performed. A standard curve was included in every run, using 1 genome equivalent (GE)/μl to 10^3^ GE/μl of B. pseudomallei (clinical isolate 1106a from Thailand; assumed genome size, 7.25 Mb). Nontemplate controls were added to each run and were always negative; i.e., no amplification was detected. Extraction controls (*n* = 10) using molecular-grade water (AccuGENE molecular biology water; Lonza) were used to rule out B. pseudomallei contamination of reagents and equipment (27). Samples with threshold cycle (*C_T_*) values below 40 were considered positive (28). The limit of detection and the specificity of the assay under the conditions described above were confirmed using B. pseudomallei (*n* = 17), B. thailandensis (*n* = 6), and B. cepacia (*n* = 11) isolates and other competing soil flora (Table 1), artificial soil (29) (*n* = 4), as well as soil from an area where melioidosis is not endemic (Germany, *n* = 4; latitude, 48°21′56.29″N; longitude, 10°50′38.32″E; altitude, 514 m).

**TABLE 1:** Isolates used to test the specificity of the molecular methods

Organism	Source	Location	Strain
International reference strains			
B. cepacia	Environmental	NA^*a*^	NCTC 10743
B. thailandensis	Environmental	Thailand	E264 (ATCC 700388)
Ralstonia pickettii	Human	Unknown	ATCC 27511
Local isolates			
B. pseudomallei (*n* = 9)	Human	Laos	Clinical isolate^*b*^
B. pseudomallei (*n* = 8)	Environment	Laos	Soil isolate^*b*^
B. cepacia (*n* = 9)	Human	Laos	Clinical isolate^*b*^
B. cepacia (*n* = 2)	Environmental	Laos	Soil isolate^*b*^
Local competing soil flora^*c*^			
Ralstonia spp. (*n* = 2)	Environmental	Vientiane, Laos	Soil isolate^*b*^^,^^*d*^
Burkholderia spp. (*n* = 12)	Environmental	Vientiane and Luang Namtha, Laos	Soil isolate^*b*^^,^^*d*^

a NA, not available.

b Identities were confirmed by determination of the colony morphology by use of the API 20NE system (bioMérieux, France) and molecular methods (36).

c Isolates collected during previous environmental surveys.

d Identities were confirmed by use of the API 20NE system (bioMérieux, France) and 16S rRNA sequencing (GenBank accession numbers KM058066 to KM058079).

### Data analysis.

For the final analysis of the data, the results of all culture methods from soil were combined, and the rate of positivity by any culture method was compared with the rate of positivity by direct DNA extraction or DNA extraction postenrichment. Statistical analysis was performed using Stata/IC (v10) software (StataCorp, College Station, TX, USA). Comparisons were made by the use of McNemar's test (paired samples) or the Mann-Whitney U test, as appropriate. Significance was set at a *P* value of <0.05.

### Nucleotide sequence accession numbers.

Sequences were analyzed using NCBI-BLAST and subsequently submitted to GenBank and are accessible under accession numbers KM058066 to KM058079 (Table 1).

## RESULTS

### Comparison of methods for B. pseudomallei detection in soil.

The qPCR was highly specific (100%) when tested with a range of reference strains and Lao clinical and environmental isolates (Table 1). The local limit of detection (LOD), which was determined using serial dilutions, was 8 GE/μl of soil DNA (equivalent to 32 GE/reaction).

**FIG 2:**
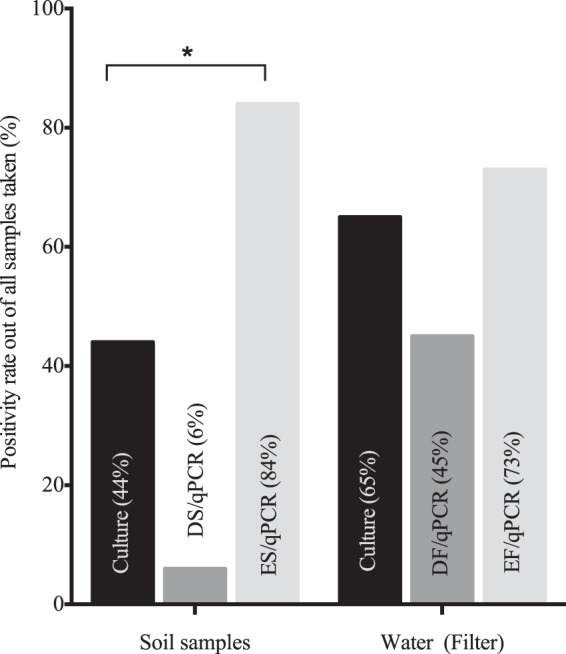
Comparison of percent positivity by detection type for different environmental sample types. The detection capacities for culture and for enrichment, DNA extraction from soil, and qPCR were significantly different (*, *P* < 0.001). The results obtained by all different culture methods for the different samples were combined.

Using the various culture methods, B. pseudomallei was detected in 44 out of 100 soil samples (44%; see Table S1 in the supplemental material). B. pseudomallei was detected in 6 out of 100 soil samples (6%) using DS DNA extraction, which was a significantly smaller number of soil samples than the number in which B. pseudomallei was detected by culture methods (*P* < 0.001). However, when using the ES DNA extraction method followed by qPCR, nearly double the number of samples (84/100, 84%) were identified to be positive compared to the number positive by the culture approach (*P* < 0.001; Fig. 2). Samples positive after direct extraction from soil contained between ∼1 and 6 × 10^2^ GE/g soil (range; median, ∼1.3 × 10^2^ GE/g soil). Of the 44 culture-positive soil samples, all but 1 were also positive by qPCR following ES DNA extraction (97.7%). No inhibition was observed with any of the extraction methods (change in *C_T_* value, ≥2), suggesting the complete removal of inhibitors using the commercial soil extraction kit. There was no difference in the overall rates of positivity by PCR and/or culture between samples taken at 30 or 60 cm (for samples taken at 60 cm, 42/50 samples [84%] were positive; for samples taken at 30 cm, 43/50 samples [86%] were positive).

**FIG 3:**
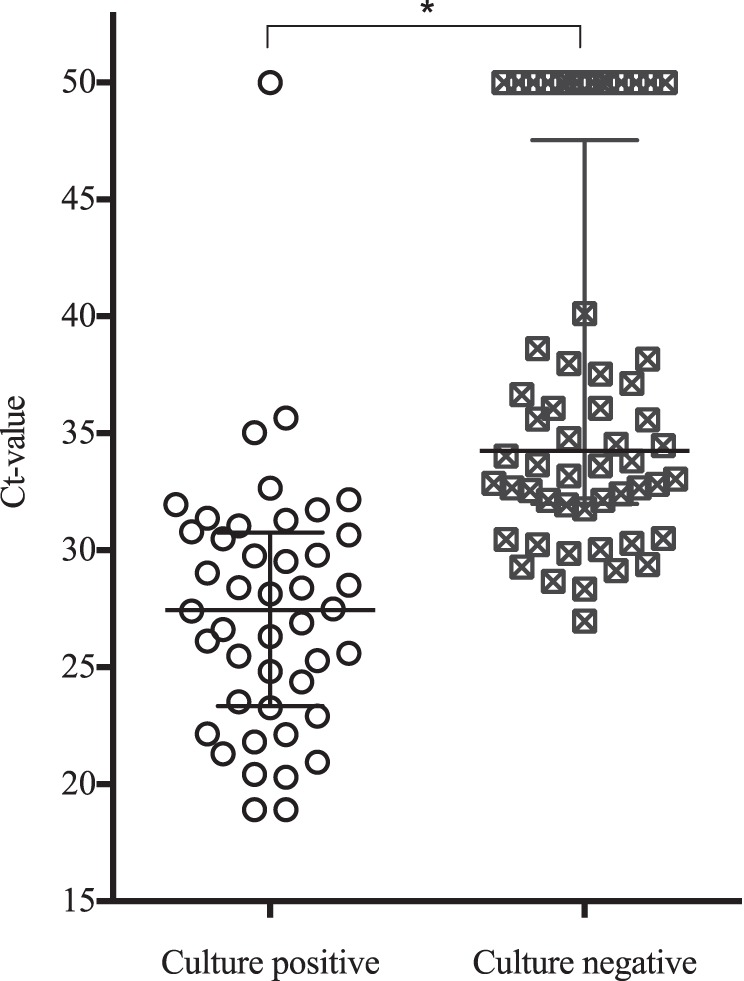
*C_T_* values of samples positive or negative by culture methods. Low *C_T_* values indicate a higher bacterial number in the template, while higher *C_T_* values are indicative of lower bacterial numbers. For illustrative purposes, samples negative by qPCR were assigned a *C_T_* value of 50. The median and interquartile ranges are marked. *, statistically significant difference (*P* < 0.001).

Due to the culture step, reliable and accurate quantification of B. pseudomallei by qPCR following enrichment was not possible. However, it was observed that culture-positive soil samples had significantly lower *C_T_* values after enrichment than culture-negative soil samples (median *C_T_* value for culture-positive soil samples = 27.4; interquartile ratio [IQR] of *C_T_* values = 23.4 to 30.7; median *C_T_* value for culture-negative soil samples = 34.3; IQR of *C_T_* values = 32.0 to 45.1; *P* = 0.0001; Fig. 3).

### Comparison of methods for B. pseudomallei detection in river water.

Overall, samples taken at 15/20 (75%) sampling points were positive by culture and/or PCR (Table 2; see also Table S2 in the supplemental material). The yield obtained by direct placement of filters on Ashdown's agar (13/20, 65%) was higher than that obtained from broth enrichment cultures (10/20, 50%); the duration of broth enrichment prior to subculture made no difference to the yield. The yield of the 0.2-μm-pore-size filter (12/20, 60%) was higher than that of the 3-μm-pore-size filter (6/20, 30%) when placed on agar; this difference was, however, not significant (*P* = 0.070). No difference in yields from filters of the two pore sizes was observed when broth enrichment was used. Only in one case was culture of the filter directly on Ashdown's agar negative while the enrichment culture was positive with a sample from the same sampling site. In total, 55% (11/20) of sampling points were positive by qPCR when DNA was directly extracted from any filter and 75% (15/20) of sampling points were positive by qPCR when the enrichment approach was used. Samples from nearly all qPCR-positive sites (14/15) were positive with both filter sizes when using the molecular approach with enrichment, with only 1 additional sample being positive with the 3-μm-pore-size filter. No difference in the positivity rates by sampling point was found when the molecular and culture methods were compared (for enrichment and qPCR, 15/20 [75%] samples were positive; for culture, 15/20 [75%] samples were positive). When comparing rates of positivity for individual filters, qPCR postenrichment was more often positive than culture methods (for qPCR postenrichment versus direct plating, *P* = 0.003; for qPCR postenrichment versus broth enrichment culture, *P* < 0.001).

**TABLE 2:** Overview of detection methods and positivity frequency for individual water samples and sampling sites^*a*^

Technique	Positivity frequency^*b*^			
	After filtering through:		In water	By sample site
	A 0.2-μm-pore-size filter	A 3-μm-pore-size filter		
Culture techniques				
Moore's swab	NA	NA	2/10 (20)	2/10 (20)
Filtering, direct plating of filter on Ashdown's agar^*c*^	12/20 (60)	6/20 (30)	NA	13/20 (65)
Filtering, filter placement in Ashdown's broth (3 days)^*c*^^,^^*d*^	6/20 (30)	6/20 (30)	NA	10/20 (50)
Filtering, filter placement in Ashdown's broth (7 days)^*c*^^,^^*d*^	6/20 (30)	6/20 (30)	NA	10/20 (50)
Molecular techniques				
Enrichment, qPCR	14/20 (70)	15/20 (75)	NA	15/20 (75)
Direct qPCR	8/20 (40)	10/20 (50)	NA	11/20 (55)

a The positivity of individual filters, as well as the overall positivity by sampling site, is listed for the individual techniques.

b Data represent the number of samples positive/total number of samples tested (percent). NA, not applicable.

c Combined observations after 48 h, 72 h, and 96 h.

d Combined results for culture of 10-μl and 100-μl supernatants.

The B. pseudomallei bacterial load was estimated to be ∼7.5 × 10^3^ GE/liter river water (median; range, 8 × 10^2^ to 1.3 × 10^5^ GE/liter river water) after direct DNA extraction from filter membranes (pore sizes, 0.2 μm and 3 μm).

## DISCUSSION

In order to increase the awareness of health care staff, assist with patient management, and implement public health actions, the availability of a detailed risk map for melioidosis is essential. Our aim was to update and improve detection methods to facilitate further research into the distribution of B. pseudomallei in the Lao environment.

Bacterial culture methods have, until now, been the methods of choice to detect B. pseudomallei in soil (9, 11, 20, 30). In this investigation, B. pseudomallei was detected in 44% of the Lao soil samples tested by culture, while DNA detection following an enrichment step gave a much higher positivity rate for the same sample set (84%). Use of an initial propagation step significantly increased the ability to detect the bacterium in Lao soil, consistent with findings from Australia (14), where DNA detection directly from soil lacked sensitivity. Direct DNA detection from clay-rich soils, like those found in the Lao rice paddy investigated in the present study (∼30 to 45% clay-size particles; data not shown), can be extremely challenging, as clay particles can reduce the extraction efficiency (14, 31) of commercial kits (32). It is therefore important to include inhibition controls to identify one important cause of false-negative results for samples. Despite the limitations when extracting DNA directly from soil, B. pseudomallei DNA has successfully been obtained from sandy loam soils from northeastern Thailand (15) using the SoilMaster DNA extraction kit (Epicentre Biotechnologies, USA). Interestingly, in that investigation the estimated bacterial counts per gram of soil were up to 140 times higher than those in Lao samples from this study (in Thailand the median is 1.8 × 10^4^ GE/g soil [15]; in Laos the median is 1.3 × 10^2^ GE/g soil). This overall lower B. pseudomallei load in Lao soil could also be responsible for the increased difficulties of bacterial DNA detection, and the observed correlation between low *C_T_* values after enrichment and culture positivity might point toward lower B. pseudomallei loads in Lao soil.

To reduce the influence of sample variations on the method comparison, we employed a rigorous soil subsampling approach (21, 33, 34). Still, the possibility that variations in results are due to an uneven distribution of the organism within the samples cannot be ruled out (33). Quantitative real-time PCR assays are thought to be highly specific, and the findings of extensive analytical specificity studies performed in this and previous studies (14, 16) support this. In addition, nontemplate and negative controls, used to monitor the potential contamination of reagents and equipment, were consistently negative, supporting the specificity of our results. In future studies, the inclusion of dedicated extraction controls monitoring the DNA extraction efficiency should be considered to further increase the quality of the data.

As with B. pseudomallei detection from soil, nucleic acid extraction from filters following an enrichment step proved to be the most sensitive and reliable method to detect the organism in water samples. This report represents the first description of the use of molecular methods to detect B. pseudomallei in surface water samples. Water sampling campaigns might represent a promising alternative to large-scale soil sampling campaigns, for example, by using river water as an initial screen to determine whether B. pseudomallei is present in the relevant catchment area (12). Despite the overall high positivity rate obtained when using qPCR postenrichment, only the combination of molecular and culture techniques allowed the identification of a B. thailandensis isolate that cross-reacted with the B. pseudomallei latex reagent (35) due to the presence of a B. pseudomallei-like extracellular exopolysaccharide (EPS), emphasizing that one methodology does not fit all research questions.

In conclusion, molecular detection methods using an additional initial enrichment step have proven to be sensitive, specific, and reliable approaches for the detection of B. pseudomallei in environmental samples, particularly soil samples. They have the potential for simple scale-up and are less time- and labor-intensive than culture methods. However, the consumables are more expensive (∼$9 per sample) than the consumables used for identification by agglutination assay alone (∼$0.50 per sample), and further follow-up phylogenetic investigations are limited without isolation of the organism.

Additional studies are needed to investigate in particular the water sampling approach and explore the influence of physical and chemical characteristics as well as bacterial loads on the different B. pseudomallei detection methods. These data suggest that molecular methods will be an important tool in establishing a melioidosis risk map in Laos and elsewhere.

## Supplementary Material

Supplemental material
