# Supplementary material for: Evaluation of Molecular Methods To Improve the Detection of Burkholderia pseudomallei in Soil and Water Samples from Laos
Source: Appl Environ Microbiol. 2015 May 5;81(11):3722–7. doi: 10.1128/AEM.04204-14 (PMC4421066; doi:10.1128/AEM.04204-14)
Supplement: Supplemental material [file supp_81_11_3722__index.html]

Evaluation of Molecular Methods To Improve the Detection of Burkholderia pseudomallei in Soil and Water Samples from Laos — Supplemental material 

# Evaluation of Molecular Methods To Improve the Detection of Burkholderia pseudomallei in Soil and Water Samples from Laos

## Supplemental material

**Files in this Data Supplement:**

- Supplemental file 1 -

  Overview of the soil sample culture and molecular results by sampling point (Table S1); overview of water characteristics and culture and molecular results by sampling point (Table S2).

  PDF, 144K
