# Supplementary material for: Evaluation of Molecular Methods To Improve the Detection of Burkholderia pseudomallei in Soil and Water Samples from Laos
Source: Appl Environ Microbiol. 2015 May 5;81(11):3722–7. doi: 10.1128/AEM.04204-14 (PMC4421066; doi:10.1128/AEM.04204-14)
Supplement: Supplemental material [file AEM.04204-14_zam999116301so1.pdf]

Table S1: Overview of the soil sample culture and molecular results by sampling point.

Sample ID	Conventional Culture	Simplified method	ES/qPCR	DS/qPCR
1.30	Positive	Positive	Positive	Negative
1.60	Positive	Negative	Positive	Positive
2.30	Positive	Positive	Positive	Positive
2.60	Positive	Negative	Positive	Negative
3.30	Positive	Negative	Positive	Negative
3.60	Positive	Positive	Positive	Negative
4.30	Negative	Negative	Positive	Negative
4.60	Negative	Negative	Positive	Negative
5.30	Negative	Negative	Positive	Negative
5.60	Positive	Negative	Positive	Negative
6.30	Positive	Negative	Positive	Negative
6.60	Positive	Negative	Positive	Negative
7.30	Positive	Negative	Positive	Negative
7.60	Positive	Negative	Positive	Positive
8.30	Positive	Positive	Positive	Positive
8.60	Positive	Positive	Positive	Negative
9.30	Positive	Positive	Positive	Negative
9.60	Negative	Negative	Positive	Negative
10.30	Positive	Negative	Positive	Negative
10.60	Negative	Positive	Positive	Negative
11.30	Negative	Negative	Positive	Negative
11.60	Negative	Negative	Positive	Negative
12.30	Negative	Negative	Positive	Negative
12.60	Negative	Negative	Negative	Negative
13.30	Positive	Negative	Positive	Negative
13.60	Positive	Negative	Positive	Negative
14.30	Negative	Negative	Positive	Negative
14.60	Negative	Negative	Positive	Negative
15.30	Negative	Negative	Positive	Negative
15.60	Negative	Negative	Positive	Negative
16.30	Negative	Negative	Positive	Negative
16.60	Negative	Negative	Negative	Negative
17.30	Negative	Negative	Positive	Negative
17.60	Negative	Negative	Positive	Negative
18.30	Positive	Negative	Positive	Negative
18.60	Negative	Negative	Negative	Negative
19.30	Negative	Negative	Positive	Negative
19.60	Positive	Positive	Positive	Positive
20.30	Negative	Negative	Positive	Negative
20.60	Negative	Negative	Positive	Negative
21.30	Negative	Negative	Negative	Negative

21.60	Negative	Negative	Positive	Negative
22.30	Positive	Negative	Positive	Negative
22.60	Negative	Negative	Positive	Negative
23.30	Positive	Negative	Positive	Negative
23.60	Positive	Positive	Positive	Negative
24.30	Positive	Negative	Positive	Negative
24.60	Negative	Negative	Positive	Negative
25.30	Negative	Negative	Negative	Negative
25.60	Negative	Negative	Negative	Negative
26.30	Negative	Negative	Negative	Negative
26.60	Positive	Negative	Positive	Negative
27.30	Negative	Negative	Positive	Negative
27.60	Positive	Negative	Positive	Negative
28.30	Positive	Negative	Positive	Negative
28.60	Positive	Negative	Positive	Negative
29.30	Positive	Negative	Positive	Negative
29.60	Positive	Negative	Positive	Negative
30.30	Positive	Negative	Positive	Negative
30.60	Positive	Positive	Positive	Negative
31.30	Negative	Negative	Negative	Negative
31.60	Positive	Negative	Positive	Negative
32.30	Negative	Negative	Positive	Negative
32.60	Positive	Negative	Positive	Negative
33.30	Negative	Negative	Positive	Negative
33.60	Positive	Negative	Positive	Negative
34.30	Positive	Negative	Positive	Negative
34.60	Positive	Negative	Negative	Negative
35.30	Negative	Negative	Negative	Negative
35.60	Negative	Negative	Positive	Negative
36.30	Negative	Negative	Positive	Negative
36.60	Negative	Negative	Positive	Negative
37.30	Negative	Negative	Positive	Negative
37.60	Negative	Negative	Negative	Negative
38.30	Negative	Negative	Positive	Negative
38.60	Negative	Negative	Positive	Negative
39.30	Negative	Negative	Positive	Negative
39.60	Negative	Negative	Positive	Negative
40.30	Negative	Negative	Negative	Negative
40.60	Positive	Negative	Positive	Negative
41.30	Negative	Negative	Positive	Negative
41.60	Negative	Negative	Negative	Negative
42.30	Negative	Negative	Positive	Negative
42.60	Positive	Negative	Positive	Negative
43.30	Negative	Negative	Positive	Negative
43.60	Negative	Negative	Positive	Negative

44.30	Negative	Negative	Positive	Negative
44.60	Positive	Negative	Positive	Negative
45.30	Positive	Negative	Positive	Positive
45.60	Positive	Negative	Positive	Negative
46.30	Negative	Negative	Positive	Negative
46.60	Positive	Negative	Positive	Negative
47.30	Positive	Negative	Positive	Negative
47.60	Negative	Negative	Positive	Negative
48.30	Negative	Negative	Positive	Negative
48.60	Negative	Negative	Positive	Negative
49.30	Negative	Negative	Positive	Negative
49.60	Negative	Negative	Negative	Negative
50.30	Negative	Negative	Negative	Negative
50.60	Negative	Negative	Negative	Negative

Table S2: Overview of water characteristics and culture and molecular results by sampling point.
Temperature was measured in 'degrees Celsius' (°C), the pH was measured as 'log protons activity' and the water turbidity was measured in nephelometric turbidity units (NTU).

Sampling station	Temperature (°C)	pH	Turbidity (NTU)	Direct culture	Enrichment culture	Culture all	EF/qPCR	DF/qPCR
SR_1	25.27	6.32	727	1	0	1	1	0
ST_1	28.24	6.75	319	1	0	1	1	1
SR_2	25.67	6.64	542	1	0	1	1	1
SR_3	25.92	6.42	446	1	0	1	1	1
ST_12	26.36	6.53	422	1	1	1	1	1
ST_11	23.88	7.32	25.9	1	1	1	1	0
ST_10	24.3	7.11	18.2	0	0	0	0	0
SR_6	26.86	7.28	177	0	0	0	0	0
ST_13	27.17	7.57	118	1	0	1	0	0
ST_4	26.88	7.41	55.9	0	0	0	0	0
SR_5	26.67	7.41	271	1	1	1	1	1
ST_3	28.16	7.07	404	0	0	0	0	0
SR_4	26.86	7.22	362	0	0	1	1	0
ST_2	28.18	5.27	585	1	1	1	1	1
SR_7	26.63	7.1	313	1	1	1	1	1
ST_6	26.24	7.74	237	1	1	1	1	1
ST_7	23.89	7.63	54.4	1	1	1	1	1
ST_8	25.33	7.82	45.9	1	1	1	1	1
ST_9	24.44	7.39	73.7	0	1	1	1	1
ST_14	27.16	7.49	417	1	1	1	1	0
